# Valor Prognóstico da Imagem de Ressonância Magnética Cardíaca de Perfusão em Estresse com Adenosina em Idosos com Doença Arterial Coronariana Conhecida ou Suspeita

**DOI:** 10.36660/abc.20210530

**Published:** 2022-07-07

**Authors:** Yodying Kaolawanich, Thananya Boonyasirinant

**Affiliations:** 1 Mahidol University Faculty of Medicine Siriraj Hospital Department of Medicine Bangkok Tailândia Division of Cardiology, Department of Medicine, Faculty of Medicine Siriraj Hospital, Mahidol University, Bangkok – Tailândia

**Keywords:** Adenosina, Ressonância Magnética Cardíaca, Doença Arterial Coronariana, Idoso, Teste de Stress

## Abstract

**Fundamento:**

Há dados limitados sobre o valor prognóstico da ressonância magnética cardíaca (RMC) em estresse em pacientes idosos.

**Objetivo:**

Determinar o valor prognóstico da RMC em estresse com adenosina em idosos com doença arterial coronariana (DAC) conhecida ou suspeita.

**Métodos:**

Entre 2010 e 2015, pacientes consecutivos com 65 anos ou mais encaminhados para RMC em estresse com adenosina foram acompanhados para a ocorrência de eventos cardíacos graves (morte cardíaca e infarto do miocárdio não-fatal) e eventos cardiovasculares adversos maiores (ECAM) que também incluíram hospitalização por insuficiência cardíaca e acidente vascular cerebral isquêmico. As análises univariadas e multivariadas foram realizadas para determinar o valor prognóstico da isquemia miocárdica, com valor de p <0,05 considerado estatisticamente significante.

**Resultados:**

Após um período médio de seguimento de 50,4 meses em 324 pacientes (48% do sexo masculino, 73±7 anos), ocorreram 21 eventos cardíacos graves e 52 ECAM. Pacientes com isquemia miocárdica (n=99) apresentaram taxas significantemente maiores de eventos cardíacos graves (HR 5,25 [IC 95% 2,11-13,04], p<0,001) e ECAM (HR 3,01 [IC 95% 1,75-5,20], p<0,001) do que aqueles sem isquemia. A análise multivariada determinou a isquemia como preditor independente de eventos cardíacos graves (HR 3,14 [IC 95% 1,22-8,07], p=0,02) e ECAM (HR 1,91 [IC 95% 1,02-3,59], p=0,04). A isquemia forneceu um valor prognóstico incremental sobre fatores clínicos e fração de ejeção do ventrículo esquerdo para predizer eventos cardíacos graves e ECAM (p<0,01 para ambos). Nenhum evento adverso grave ocorreu durante ou imediatamente após os exames de RMC.

**Conclusão:**

A RMC em estresse com adenosina é segura e demonstra valor prognóstico em idosos com DAC conhecida ou suspeita.

## Introdução

O envelhecimento está associado a alterações difusas em todo o sistema cardiovascular. A prevalência e a gravidade da doença arterial coronariana (DAC) aumentam progressivamente com a idade em homens e mulheres.^1^ Nos países desenvolvidos, aproximadamente dois terços de todos os infartos do miocárdio (IM) ocorrem em pessoas com mais de 65 anos.^2^ Os idosos têm maior probabilidade de apresentar sintomas atípicos, como falta de ar ou fadiga por esforço, em vez de angina típica.^3^ A prevalência de isquemia miocárdica silenciosa e infarto do miocárdio (IM) não reconhecido também é significativamente maior em idosos e tem valor prognóstico.^4^ Pacientes mais velhos também tendem a ter um risco aumentado de complicações, incluindo insuficiência cardíaca, arritmias, sangramento e morte no cenário de procedimentos cardíacos, como intervenção coronária percutânea ou cirurgia cardíaca. Portanto, o diagnóstico e a estratificação de risco de DAC em pacientes idosos são extremamente importantes.

O teste de isquemia em pacientes idosos é um desafio. O teste de esforço é menos viável em idosos devido à menor capacidade de exercício e comorbidades, bem como anormalidades eletrocardiográficas (ECG) de base que limitam as avaliações isquêmicas. A ressonância magnética cardíaca (RMC) fornece uma avaliação abrangente da DAC com altíssima precisão. A RMC pode avaliar a função ventricular global e regional, isquemia miocárdica e infarto em um único estudo. Além disso, a RMC em estresse farmacológico oferece fortes evidências para o prognóstico, incluindo a mortalidade em pacientes com DAC conhecida ou suspeita.^5–8^

Dados anteriores mostraram que a RMC de perfusão em estresse realizada em pacientes idosos é segura e bem tolerada.^9,10^ Um estudo recente relatou o valor prognóstico da RMC de perfusão em estresse com dipiridamol em pacientes idosos com suspeita de DAC.^10^ A adenosina é mais frequentemente utilizada para RMC de perfusão em estresse na prática clínica. Entretanto, os dados prognósticos da RMC em estresse com adenosina em pacientes idosos permanecem limitados.

O objetivo deste estudo foi determinar o valor prognóstico da RMC em estresse com adenosina em idosos com DAC conhecida ou suspeita.

## Métodos

### População do estudo

Pacientes consecutivos com mais de 65 anos com DAC conhecida ou suspeita que foram encaminhados para RMC em estresse com adenosina de janeiro de 2010 a dezembro de 2015 em nosso ambulatório foram recrutados. O histórico médico detalhado foi coletado no mesmo dia do exame de RMC. O histórico de hipertensão, diabetes mellitus, hiperlipidemia, DAC e acidente vascular cerebral foram definidos por diretrizes recentes.^11–14^

Os critérios de exclusão incluíram (i) miocardiopatia não-isquêmica conhecida (por exemplo, hipertrófica, dilatada ou infiltrativa), (ii) exame de RMC incompleto, (iii) imagens de RMC de má qualidade e (v) falta de dados de seguimento. O comitê de ética institucional aprovou este estudo retrospectivo e dispensou a necessidade de consentimento informado por escrito adicional.

Tem havido preocupação quanto à associação do uso de gadolínio com o desenvolvimento de fibrose sistêmica nefrogênica em pacientes com insuficiência renal grave, especialmente em idosos. Os pacientes que apresentaram taxa de filtração glomerular <30 mL/min/1,73m^2^ não foram submetidos ao exame de RMC com contraste e não foram incluídos neste estudo.^15^

### Protocolo da RMC

O estudo da RMC foi realizado para avaliar a função cardíaca, perfusão miocárdica e realce tardio de gadolínio (RTG) utilizando um scanner Philips Achieva XR de 1,5 Tesla (Philips Medical Systems, Best, Países Baixos).

O estudo funcional cardíaco foi realizado através da aquisição das imagens utilizando a técnica de precessão livre no estado estacionário (SSFP, *steady-state free precession*) em cortes verticais de eixo longo, de 2 câmaras, 4 câmaras e múltiplos cortes de eixo curto. Os parâmetros para a função cardíaca foram tempo de eco (TE) 1,8 milissegundos (ms), tempo repetitivo (TR) 3,7 ms, número de excitações 2, campo de visão (FOV, *field of view*) 390 × 312 mm, matriz 256 × 240, pixels de reconstrução 1,52 × 1,21, espessura da lâmina de 8 mm e ângulo de inclinação de 70 graus.

O estudo de perfusão miocárdica de primeira passagem foi realizado por injeção de 0,05 mmol/kg de agente de contraste de gadolínio (Magnevist, Bayer Schering Pharma, Berlim, Alemanha) a uma taxa de 4 mL/s imediatamente após uma infusão de 4 minutos de 140 mcg/kg/min de adenosina.^16^ Se após 3 minutos de infusão contínua na taxa padrão a resposta hemodinâmica à adenosina fosse inadequada (aumento da frequência cardíaca <10 batimentos/min ou diminuição da pressão arterial sistólica <10 mmHg, com mínimo ou nenhum efeito colateral relatado pelo paciente), então a taxa de infusão foi aumentada até 210 mcg/kg/min por mais 2 minutos.^16^ Três cortes de eixo curto nos níveis do ventrículo esquerdo (VE) basal, médio e apical foram adquiridos utilizando um ECG de disparo (ECG-*triggered*), SSFP, sequência de inversão-recuperação, *single-shot*, turbo gradiente eco. Os parâmetros de imagem foram TE 1,32 ms, TR 2,6 ms, ângulo de inversão de 50 graus, espessura de corte de 8 mm, FOV 270 mm e FOV reconstruído 320 mm.

As imagens de RTG foram adquiridas aproximadamente 10 minutos após um bolus adicional de gadolínio (0,1 mmol/kg, taxa de 4 mL/s) pela sequência 3D segmentado-gradiente-eco inversão-recuperação. As imagens de RTG foram adquiridas em múltiplos cortes de eixo curto em níveis semelhantes às imagens funcionais, eixo longo, visão de 2 câmaras e 4 câmaras. Os parâmetros para o estudo com RTG foram TE 1,25 ms, TR 4,1 ms, ângulo de inclinação 15 graus, FOV 303 × 384 mm, matriz 240 × 256, resolução no plano 1,26 × 1,5 mm, espessura de corte 8 mm e fator de codificação de sensibilidade de 1,5.

### Análise de imagem

Volumes padrão do VE, massa e fração de ejeção (FE) foram medidos quantitativamente a partir da pilha de imagens cine SSFP de eixo curto.

As imagens de perfusão e RTG foram analisadas utilizando avaliação visual e consenso por dois médicos treinados em RMC, cegos para dados clínicos e de seguimento. As imagens de perfusão foram lidas e cada um dos 16 segmentos foi visualizado (segmento-17 no ápice não foi visualizado). A isquemia induzível foi definida como um defeito de perfusão subendocárdica que (i) persistiu além do pico de realce miocárdico e por vários intervalos RR, (ii) tinha mais de dois pixels de largura, (iii) seguia uma ou mais artérias coronárias e (iv) mostrava ausência de RTG no mesmo segmento.^10,17^ Artefatos de banda escura eram registrados se uma banda escura endocárdica aparecesse na chegada do contraste na cavidade do VE antes da chegada do contraste no miocárdio.^17^ As imagens de RTG também foram analisadas por avaliação visual. O RTG foi considerado presente apenas se confirmado no eixo curto e em pelo menos um outro plano ortogonal.^17^ O número total de segmentos do RTG foi calculado com o modelo de 17 segmentos da *American Heart Association*.^18^

### Seguimento clínico

Os dados de seguimento foram coletados das visitas clínicas e prontuários médicos. A adjudicação de eventos clínicos foi completamente mascarada para dados clínicos e da RMC. Os pacientes foram acompanhados para a ocorrência de eventos cardíacos graves e eventos cardiovasculares adversos maiores (ECAM). Eventos cardíacos graves foram definidos como os desfechos compostos de mortalidade cardíaca e IAM não fatal.^19^ Os ECAM foram definidos como os desfechos compostos de mortalidade cardíaca, IAM não fatal, hospitalização por insuficiência cardíaca e acidente vascular cerebral isquêmico.

### Análise estatística

As análises estatísticas foram realizadas utilizando o *software* IBM SPSS *Statistics for Windows*, versão 20.0 (IBM Corp., Armonk, NY, EUA). As variáveis contínuas com distribuição normal foram apresentadas como média ± desvio padrão (DP). A normalidade da distribuição das variáveis foi avaliada pelo teste de Kolmogorov-Smirnov. As variáveis categóricas foram apresentadas como números absolutos e percentuais. As diferenças entre os pacientes com e sem isquemia miocárdica em relação à linha basal clínica e às características da RMC foram comparadas usando o teste *t* não pareado de Student para variáveis contínuas e o teste qui-quadrado ou teste exato de Fisher para variáveis categóricas, como apropriado.

Os desfechos compostos entre pacientes com e sem isquemia miocárdica foram estimados pelo método de Kaplan-Meier e comparados com o teste de *log-rank*. Para analisar os preditores de eventos cardíacos graves e ECAM, uma análise de regressão de Cox foi realizada para avaliar preditores univariáveis de características basais e parâmetros da RMC. Variáveis com valor de p <0,05 na análise univariável foram inseridas na análise multivariável. Dois modelos multivariáveis foram desenvolvidos para avaliar o valor prognóstico da isquemia miocárdica. Primeiramente, a isquemia foi incluída como variável categórica (presença ou ausência). Em segundo lugar, a isquemia foi incluída como variável contínua (extensão por segmento).

Para avaliar os valores de prognóstico incremental de preditores significativos, os valores globais do qui-quadrado foram calculados após a adição de preditores na seguinte ordem: dados clínicos, FEVE, isquemia e RTG.

Foram calculadas as *hazard ratios* (HRs) e intervalos de confiança de 95% (ICs) dos desfechos, com valor de p < 0,05 sendo considerado estatisticamente significante.

## Resultados

Um total de 327 pacientes foram inscritos, com três excluídos devido à perda de dados de seguimento. Nenhum paciente foi excluído devido à má qualidade da imagem e 324 foram incluídos na análise final. A Tabela 1 resume os dados clínicos da população de pacientes. A média de idade foi de 73±7 anos. Quarenta e seis pacientes tinham DAC conhecida e 6 tinham IM prévio. A coorte geral do estudo apresentou FEVE média de 68,8±13,8%.

**Tabela 1:** Características clínicas dos pacientes com e sem isquemia miocárdica

	Total	Isquemia Presente	Isquemia Ausente	Valor de p
	(n=324)	(n=99)	(n=225)	
Sexo masculino	156 (48,1)	55 (55,6)	101 (44,9)	0,08
Idade, anos	72,7 ± 7,4	72,9 ± 7,7	72,6 ± 7,3	0,73
Índice de massa corporal, kg/m^2^	26,5 ± 4,2	25,8 ± 3,9	26,9 ± 4,2	**0,03**
Pressão arterial sistólica, mmHg	138,8 ± 18,9	142,2 ± 19,3	137,3 ± 18,7	**0,03**
Pressão arterial diastólica, mmHg	72,8 ± 11,5	71,9 ± 12,1	73,2 ± 11,2	0,33
Frequência cardíaca, bpm	76,9 ± 13,1	76,2 ± 12,8	77,2 ± 13,3	0,52
Hipertensão	289 (89,2)	87 (87,8)	202 (89,8)	0,61
Diabetes mellitus	188 (58,0)	57 (57,6)	131 (58,2)	0,91
Hiperlipidemia	231 (71,3)	74 (74,8)	157 (69,8)	0,36
DAC estável	46 (14,2)	28 (28,3)	18 (8,0)	**<0,001**
Infarto do miocárdio prévio	6 (1,9)	5 (5,1)	1 (0,4)	**0,01**
Revascularização prévia	14 (4,3)	8 (8,1)	6 (2,7)	**0,04**
História de angina típica	31 (9,6)	15 (15,2)	16 (7,1)	**0,02**
História de insuficiência cardíaca	23 (7,1)	9 (9,1)	14 (6,2)	0,35
Acidente vascular cerebral	16 (4,9)	4 (4,0)	12 (5,3)	0,78
Tabagismo atual	37 (11,4)	22 (22,2)	15 (6,7)	<0,001
**Medicamentos**				
IECA ou ARB	148 (45,7)	50 (50,5)	98 (43,6)	0,25
Antiplaquetário	153 (47,2)	60 (60,6)	93 (41,3)	**0,001**
Betabloqueador	151 (46,6)	47 (47,5)	104 (46,2)	0,84
Bloqueador de canais de cálcio	111 (34,3)	35 (35,4)	76 (33,8)	0,78
Nitrato	49 (15,1)	25 (25,3)	24 (10,7)	**0,001**
Estatina	156 (48,2)	51 (51,5)	105 (46,7)	0,42
**RMC**				
Diâmetro do átrio esquerdo, mm	32,9 ± 4,0	33,6 ± 4,1	32,6 ± 3,9	0,05
Índice de massa do VE, g/m^2^	51,9 ± 16,8	59,0 ± 18,8	48,9 ± 14,8	**<0,001**
Índice de VDFVE, mL/m^2^	74,7 ± 24,4	82,1 ± 29,0	71,5 ± 21,4	**<0,001**
Índice de VSFVE, mL/m^2^	25,7 ± 22,9	32,2 ± 29,9	22,8 ± 18,3	**<0,001**
FEVE, %	68,8 ± 13,8	65,1 ± 17,5	70,5 ± 11,5	**0,001**
Presença de RTG	67 (20,7)	45 (45,5)	22 (9,8)	**<0,001**
Número médio de segmentos com RTG	4,1 ± 2,5	4,3 ± 2,6	3,6 ± 2,4	0,16

Os valores são números (porcentagens) ou média ± SD. Os valores em **negrito** são <0,05. IECA: inibidor da enzima conversora de angiotensina; BRA: bloqueador do receptor da angiotensina II; DAC: doença arterial coronariana; RMC: ressonância magnética cardíaca; VDF: volume diastólico final; VSF: volume sistólico final; EF: fração de ejeção; RTG: realce tardio com gadolínio; VE: ventrículo esquerdo.

A isquemia miocárdica foi detectada em 99 pacientes (31%) com número médio de segmentos isquêmicos de 6,9±3,9. Sessenta e sete tinham RTG e todos apresentavam padrão de DAC (RTG subendocárdico ou transmural). De 67 pacientes com RTG, 3 tinham histórico de IM. Portanto, 64 pacientes (19,7%) tinham RTG sem histórico de IM (‘IM não reconhecido’).

Pacientes com isquemia miocárdica apresentaram maior índice de massa do VE, menor FEVE e maior prevalência de RTG do que aqueles sem isquemia. Os pacientes com isquemia também eram mais propensos a ter um histórico de DAC ou IM e estar recebendo terapia antiplaquetária e nitrato.

Nenhum paciente morreu durante ou logo após a RMC, enquanto um caso de insuficiência cardíaca leve necessitou de ajuste de diuréticos sem internação hospitalar. Dois pacientes apresentaram angina que foi resolvida rapidamente com o uso de nitratos sublinguais. Nenhum caso de infarto agudo do miocárdio ou acidente vascular cerebral foi registrado durante ou imediatamente após a RMC. Os principais eventos adversos menores incluíram cefaleia, náusea, desconforto torácico, dispneia e queda transitória da pressão arterial.

**Figura 1:**
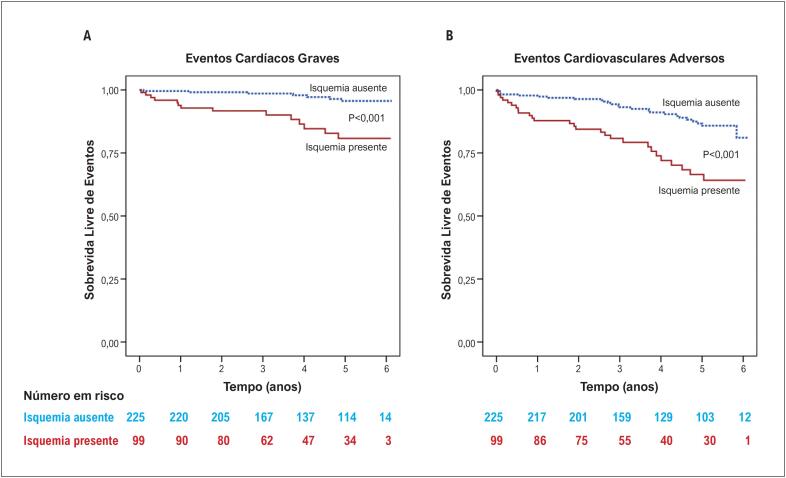
Curvas de Kaplan-Meier para a incidência de eventos cardíacos graves (A) e ECAM (B). HR: hazard ratio; ECAM: eventos cardiovasculares adversos maiores.

Durante o período médio de seguimento de 50,4±19,2 meses, ocorreram 21 eventos cardíacos graves e 52 ECAM. A Tabela 2 mostra os eventos cardiovasculares em pacientes com e sem isquemia. As curvas de Kaplan-Meier de ambos os grupos são mostradas na Figura 1. Os pacientes com isquemia miocárdica apresentaram taxas significantemente maiores de eventos cardíacos graves (taxa de eventos anuais de 3,8% versus 0,7%, p<0,001) e ECAM (taxa anual de eventos de 7,9% versus 2,7, %, p<0,001) do que aqueles sem isquemia.

**Tabela 2:** Desfechos dos pacientes

	Total	Isquemia Presente	Isquemia Ausente	HR (IC95%)	Valor de p
Mortalidade por todas as causas	18 (5,6)	10 (10,1)	8 (3,6)	3,13 (1,23, 7,94)	**0,02**
Mortalidade cardíaca	8 (2,5)	6 (6,1)	2 (0,9)	7,59 (1,53, 37,66)	**0,01**
Infarto do miocárdio não fatal	18 (5,6)	12 (12,1)	6 (2,7)	5,22 (1,95, 13,94)	**0,001**
Hospitalização por IC	31 (9,6)	16 (16,2)	15 (6,7)	2,81(1,38, 5,70)	**0,004**
AVC isquêmico	9 (2,8)	3 (3,0)	6 (2,7)	1,31 (0,32, 5,25)	0,70
Eventos cardíacos graves^a^	21 (6,5)	14 (14,1)	7 (3,1)	5,25 (2,11, 13,04)	**<0,001**
ECAM^b^	52 (16,0)	27 (27,3)	25 (11,1)	3,01 (1,75, 5,20)	**<0,001**

Eventos cardíacos graves: desfechos compostos de mortalidade cardíaca e infarto do miocárdio não fatal. ECAM: desfechos compostos de mortalidade cardíaca, infarto do miocárdio não fatal, hospitalização por insuficiência cardíaca e acidente vascular cerebral isquêmico.

a Cinco pacientes tiveram dois eventos (infarto do miocárdio não fatal e mortalidade cardíaca).

b Nove pacientes tiveram mais de um evento (seis pacientes tiveram dois eventos, um paciente teve três eventos e dois pacientes tiveram quatro eventos). Os valores representam o número de pacientes (porcentagens). Os valores em **negrito** são <0,05. IC: intervalo de confiança; HR: hazard ratio; ECAM: eventos cardiovasculares adversos maiores.

As análises univariáveis e multivariáveis para prever eventos cardíacos graves e ECAM são apresentadas nas Tabelas 3 e 4, respectivamente. O número de pacientes e eventos foi limitado; portanto, para evitar o potencial de *overfitting*, foram incluídos apenas os preditores mais significativos da análise univariável em qualquer modelo multivariável.

**Tabela 3:** Preditores de eventos cardíacos graves

	Análise Univariável		Análise multivariável			
			Modelo 1^a^		Modelo 2^b^	
	HR (95% CI)	Valor de p	HR (IC95%)	Valor de p	HR (IC95%)	Valor de p
Sexo masculino	1,26 (0,53, 2,97)	0,59				
Idade, anos	1,01 (0,95, 1,07)	0,70				
Índice de massa corporal, kg/m^2^	0,90 (0,81, 1,01)	0,08				
Pressão arterial sistólica	0,99 (0,97, 1,02)	0,63				
Pressão arterial diastólica	0,98 (0,94, 1,02)	0,33				
Frequência cardíaca, bpm	1,01 (0,97, 1,04)	0,71				
Hipertensão	2,57 (0,34, 19,17)	0,36				
Diabetes mellitus	1,21 (0,51, 2,89)	0,67				
Hiperlipidemia	1,06 (0,39, 2,92)	0,90				
DAC estável	2,26 (0,82, 6,19)	0,11				
Infarto do miocárdio prévio	9,36 (2,75, 31,81)	**<0,001**	6,70 (1,83, 24,49)	**0,004**	5,90 (1,52, 22,93)	**0,01**
Histórico de angina típica	2,80 (1,02, 7,65)	**0,04**				
Histórico de insuficiência cardíaca	2,78 (0,93, 8,30)	0,07				
Acidente vascular cerebral	0,05 (0,00-177,4)	0,46				
Tabagismo atual	1,82 (0,61, 5,41)	0,28				
IECA ou BRA	1,11 (0,46, 2,60)	0,82				
Antiplaquetário	2,09 (0,84, 5,20)	0,11				
Betabloqueador	1,15 (0,48, 2,71)	0,75				
Bloqueador de canais de cálcio	0,96 (0,38, 2,38)	0,94				
Nitrato	3,03 (1,25, 7,33)	**0,01**				
Estatina	1,46 (0,61, 3,47)	0,39				
Diâmetro do átrio esquerdo, mm	1,16 (1,06, 1,27)	**0,002**				
Índice de massa do VE, g/m^2^	1,03 (1,02, 1,05)	**<0,001**	1,04 (1,02, 1,05)	**0,001**	1,03 (1,02, 1,05)	**0,001**
Índice de VDFVE, ml/m^2^	1,02 (1,01, 1,03)	**<0,001**				
Índice VSFVE, mL/m^2^	1,02 (1,01, 1,03)	**0,001**				
FEVE, %	0,96 (0,94, 0,99)	**0,01**				
Presença de isquemia miocárdica	5,25 (2,11, 13,04)	**<0,001**	3,14 (1,22, 8,07)	**0,02**	-	-
Extensão da isquemia, por 1 segmento	1,17 (1,09, 1,26)	**<0,001**	-	**-**	1,11 (1,02, 1,20)	**0,01**
Presença de RTG	4,97 (2,11, 11,73)	**<0,001**				

a Isquemia miocárdica foi incluída como variável categórica (presença ou ausência).

b Isquemia miocárdica foi incluída como variável contínua (extensão por segmento). Os valores em **negrito** são <0.05. IECA: inibidor da enzima conversora de angiotensina; BRA: bloqueador do receptor da angiotensina II;DAC: doença arterial coronariana; RMC: ressonância magnética cardíaca; VDF: volume diastólico final; VSF: volume sistólico final; FE: fração de ejeção; RTG: realce tardio com gadolínio; VE: ventrículo esquerdo.

**Tabela 4:** Preditores de eventos cardiovasculares adversos maiores

	Análise Univariável		Análise multivariável			
			Modelo 1^a^		Modelo 2^b^	
	HR (IC95%)	Valor de p	HR (IC95%)	Valor de p	HR (IC95%)	Valor de p
Sexo masculino	1,15 (0,67, 1,99)	0,61				
Idade, anos	1,05 (1,01, 1,08)	**0,02**				
Índice de massa corporal, kg/m^2^	0,98 (0,92, 1,05)	0,60				
Pressão arterial sistólica	0,99 (0,98, 1,01)	0,43				
Pressão arterial diastólica	0,97 (0,95, 0,99)	**0,02**				
Frequência cardíaca, bpm	1,01 (0,99, 1,03)	0,30				
Hipertensão	2,11 (0,66, 6,78)	0,21				
Diabetes mellitus	1,21 (0,70, 2,11)	0,50				
Hiperlipidemia	1,17 (0,61, 2,23)	0,64				
DAC estável	1,58 (0,77, 3,24)	0,22				
Infarto do miocárdio prévio	6,13 (2,21, 17,06)	**0,001**				
Histórico de angina típica	1,43 (0,64, 3,17)	0,38				
Histórico de insuficiência cardíaca	3,70 (1,90, 7,20)	**<0,001**	3,50 (1,79, 6,82)	**0,001**	3,32 (1,70, 6,50)	**0,001**
Acidente vascular cerebral	1,15 (0,36, 3,70)	0,81				
Tabagismo atual	1,62 (0,79, 3,33)	0,19				
IECA ou BRA	1,23 (0,71, 2,11)	0,46				
Antiplaquetário	1,57 (0,90, 2,73)	0,11				
Betabloqueador	1,02 (0,59, 1,77)	0,93				
Bloqueador de canais de cálcio	0,69 (0,37, 1,27)	0,24				
Nitrato	1,87 (1,01, 3,45)	**0,04**				
Estatina	1,19 (0,69, 2,05)	0,53				
Diâmetro do átrio esquerdo, mm	1,13 (1,06, 1,20)	**<0,001**				
Índice de massa do VE, g/m^2^	1,03 (1,02, 1,04)	**<0,001**				
Índice de VDFVE, mL/m^2^	1,02 (1,01, 1,03)	**<0,001**				
Índice VSFVE, mL/m^2^	1,02 (1,01, 1,03)	**<0,001**				
FEVE, %	0,97 (0,95, 0,98)	**<0,001**				
Presença de isquemia miocárdica	3,01 (1,75, 5,20)	**<0,001**	1,91 (1,02, 3,59)	**0,04**	-	-
Extensão da isquemia, por 1 segmento	1,11 (1,06, 1,17)	**<0,001**	-	**-**	1,08 (1,01, 1,14)	**0,02**
Presença de LGE	3,70 (2,13, 6,43)	**<0,001**	2,64 (1,39, 4,99)	**0,003**	2,86 (1,58, 5,17)	**0,001**

a Isquemia miocárdica foi incluída como variável categórica (presença ou ausência).

b Isquemia miocárdica foi incluída como variável contínua (extensão por segmento). Os valores em **negrito** são <0.05. IECA: inibidor da enzima conversora de angiotensina; BRA: bloqueador do receptor da angiotensina II; DAC: doença arterial coronariana; RMC: ressonância magnética cardíaca; VDF: volume diastólico final; VSF: volume sistólico final; FE: fração de ejeção; RTG: realce tardio com gadolínio; VE: ventrículo esquerdo.

Os preditores mais significativos identificados pela análise univariável para eventos cardíacos graves foram IM prévio, índice de massa do VE, índice do volume diastólico final do VE, isquemia miocárdica e RTG (p<0,001 para todos). Histórico de insuficiência cardíaca, diâmetro do átrio esquerdo, índice de massa do VE, FEVE, isquemia miocárdica e RTG foram os preditores mais significativos para ECAM (p<0,001 para todos).

As análises multivariadas mostraram que IM prévio, índice de massa do VE e isquemia miocárdica foram preditores independentes de eventos cardíacos graves. Para os ECAMs, histórico de insuficiência cardíaca, isquemia miocárdica e RTG foram preditores independentes. Observe que tanto a presença de isquemia miocárdica (modelo 1) quanto o número de segmentos isquêmicos (modelo 2) foram preditores independentes para eventos cardíacos graves e ECAM.

**Figura 2:**
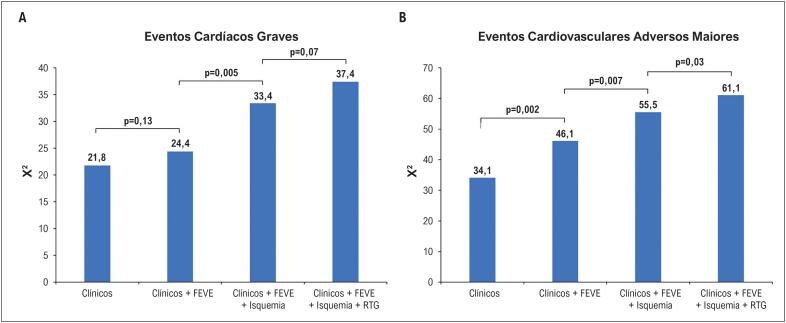
Valor prognóstico incremental da FEVE, isquemia miocárdica e RTG para eventos cardíacos graves (A) e ECAM (B). Dados clínicos=idade, sexo masculino, infarto do miocárdio prévio e histórico de insuficiência cardíaca. RTG: realce tardio com gadolínio; FEVE: fração de ejeção do ventrículo esquerdo; ECAM: eventos cardiovasculares adversos maiores.

A Figura 2 mostra valores prognósticos incrementais de dados clínicos e de RMC para prever eventos cardíacos graves e ECAM. Quando o prognóstico foi avaliado de forma hierárquica (somente variáveis clínicas, clínica+FEVE, clínica+FEVE+isquemia e clínica+FEVE+isquemia+RTG), a presença de isquemia miocárdica demonstrou valor prognóstico incremental sobre as variáveis clínicas e FEVE para ambos os eventos cardíacos graves (Figura 2A) e ECAM (Figura 2B). A adição de RTG forneceu um valor prognóstico adicional para ECAM (Figura 2B). Entretanto, o RTG não apresentou valor prognóstico incremental sobre a isquemia para eventos cardíacos graves (Figura 2A).

Dezoito pacientes morreram durante o seguimento. Dez pacientes morreram de causas não cardíacas (por exemplo, malignidade). Pacientes com isquemia miocárdica apresentaram taxa significantemente maior de mortalidade por todas as causas do que aqueles sem isquemia (Tabela 2). No entanto, não houve diferença significativa entre os pacientes com e sem isquemia quanto à taxa de mortalidade não cardíaca (HR 1,66, IC 95% 0,47-5,88, p=0,44).

## Discussão

Nossos resultados demonstraram que a isquemia miocárdica utilizando a RMC de perfusão em estresse com adenosina foi um forte e independente preditor de eventos cardíacos graves e ECAM em idosos com DAC conhecida ou suspeita. A RMC em estresse com adenosina também foi viável e segura nessa população.

A maioria das doenças cardiovasculares, incluindo a DAC, aumenta em prevalência e gravidade com a idade. O diagnóstico, a estratificação de risco e o tratamento da DAC em pacientes idosos permanecem desafiadores. A DAC estável manifesta-se de forma diferente em idosos, sendo dispneia de esforço, fadiga e desconforto abdominal as apresentações mais comuns.^3^ O envelhecimento e as comorbidades limitam a capacidade de exercício; portanto, o teste de ECG em esteira e a ecocardiografia de esforço são impraticáveis nessa população. Imagens cardíacas de estresse farmacológico, ais como imagem de perfusão nuclear e RMC, são as modalidades preferidas; no entanto, dados recentes revelaram acurácia limitada da imagem de perfusão nuclear em comparação com a RMC. Dados de grandes estudos multicêntricos sugeriram que a RMC demonstrou maior sensibilidade do que a imagem de perfusão nuclear para detecção de DAC em homens e mulheres.^20,21^ Diferente da imagem de perfusão nuclear, a RMC não expõe os pacientes à radiação ionizante e oferece precisão e segurança.

A função miocárdica global e regional é um conhecido preditor de gravidade e prognóstico da doença.^22^ A RMC é considerada o padrão-ouro para avaliação da função ventricular global e uma boa ferramenta para avaliação da função ventricular regional.^23,24^ Idosos têm uma maior prevalência de doenças pulmonares, como doença pulmonar obstrutiva crônica e isso pode limitar a avaliação pelo ecocardiograma devido a uma janela ecocardiográfica deficiente. A RMC pode avaliar a função cardíaca sem a limitação do plano cardíaco e também avaliar as bordas endocárdicas e epicárdicas sem suposições geométricas. Pacientes idosos podem ser mais vulneráveis a eventos adversos durante ou após a RMC (por exemplo, arritmia ou hipotensão) devido à alta prevalência de comorbidades. A aplicabilidade e a segurança da RMC em estresse foram determinadas em pacientes com mais de 70 anos, com resultados mostrando que a RMC em estresse realizada em pacientes idosos foi segura e bem tolerada.^9,10^ Nossos resultados confirmaram que a RMC em estresse com adenosina foi segura em idosos sem eventos adversos graves como morte, infarto agudo do miocárdio ou acidente vascular cerebral durante ou imediatamente após os exames de RMC.

Inúmeros estudos demonstraram o valor prognóstico da RMC em pacientes com DAC conhecida ou suspeita.^5–8^ Entretanto, a média de idade dos pacientes nesses estudos foi de 60-65 anos, sem avaliação específica de idosos. Pezel et al. relataram o valor prognóstico da RMC de perfusão em estresse com dipiridamol em 754 pacientes idosos acima de 75 anos com suspeita de DAC.^10^ Em seu estudo, 20% dos pacientes apresentaram evidência de isquemia induzível, enquanto 9,4% tinham RTG. Os autores determinaram que a presença de isquemia miocárdica estava associada à ocorrência de ECAM, incluindo morte cardíaca e IM não fatal.^10^ Nosso estudo, que incluiu pacientes com DAC estável conhecida e IM prévio, observou que 30,5% dos pacientes tinham isquemia induzível e 20,7% tinham RTG. A prevalência de isquemia miocárdica em nosso estudo foi comparável a relatos anteriores que incluíram pacientes com DAC conhecida.^5,7^ De maneira similar, pacientes com isquemia induzível em nosso estudo demonstraram FEVE menor e maior prevalência de RTG do que aqueles sem isquemia miocárdica.^5–7^

Nossos resultados indicaram que pacientes com isquemia induzível apresentaram taxas significantemente maiores de eventos cardíacos graves e ECAM do que aqueles sem isquemia. A isquemia miocárdica também foi um preditor independente de eventos cardíacos graves e ECAM. Em contraste, pacientes sem isquemia miocárdica apresentaram um risco significativamente menor de eventos cumulativos (<1% ao ano para eventos cardíacos graves). Esses achados concordaram com os de Pezel et al.^10^

O RTG é um método bem validado para detectar cicatrizes miocárdicas e fibrose.^25^ Padrões cicatriciais específicos correspondentes a IM e várias cardiomiopatias não isquêmicas são úteis para o diagnóstico.^25,26^ Diretrizes recentes destacaram a importância da imagem da fibrose miocárdica por RMC.^14,27^ Uma proporção significante de pacientes com DAC estável apresenta função sistólica do VE normal. A presença de RTG também demonstrou seu valor prognóstico em pacientes com FEVE e movimento da parede normais.^28^ Similar ao nosso estudo, a função sistólica do VE foi preservada. O RTG foi detectado em 20,7% dos pacientes, e foi um preditor independente de ECAM. Além disso, dada a proporção muito pequena de pacientes com histórico de IM (<2%), nossos dados também demonstraram uma prevalência compatível de ‘IM não reconhecido’ (19,7%) em comparação com dados anteriores.^3,29–33^ O IM não reconhecido não é uma condição incomum, com uma prevalência de aproximadamente 10-40% dos pacientes com DAC conhecida ou suspeita.^3,29–33^ A RMC-RTG melhorou a detecção de pequenas lesões causadas por IM (até 1 g), que não originam ondas Q no ECG.^29,30,33^ Além disso, estudos recentes demonstraram consistentemente que o IM não reconhecido utilizando RMC-LGE estava independentemente associado a um risco aumentado de eventos cardiovasculares.^29,30,33^

### Limitações

Várias limitações do presente estudo devem ser consideradas. Primeiramente, a metodologia do estudo foi retrospectiva e, portanto, alguns fatores de confusão não puderam ser totalmente eliminados. Em segundo lugar, o protocolo de estresse adquiriu apenas três cortes de eixo curto para detectar isquemia miocárdica e pode ter subestimado o defeito de perfusão em algumas áreas pequenas (em comparação com quatro ou cinco cortes de eixo curto). Em terceiro lugar, o presente estudo teve uma taxa de eventos relativamente baixa, enquanto algum grau de *overfitting* pode ter ocorrido nas análises multivariáveis. Por fim, não fornecemos informações sobre a adequação da terapia médica após a RMC em estresse que poderia afetar o prognóstico.

## Conclusões

A RMC em estresse com adenosina é segura e mostra valor prognóstico em idosos com DAC conhecida ou suspeita.

## References

[1] Mozaffarian D, Benjamin EJ, Go AS, Arnett DK, Blaha MJ, Cushman M, et al. Heart Disease and Stroke Statistics-2016 Update: A Report from the American Heart Association. Circulation. 2016;133(4):e38-360. doi: 10.1161/CIR.0000000000000350. 10.1161/CIR.0000000000000350 26673558

[2] Yazdanyar A, Newman AB. The Burden of Cardiovascular Disease in the Elderly: Morbidity, Mortality, and Costs. Clin Geriatr Med. 2009;25(4):563-77. doi: 10.1016/j.cger.2009.07.007. 10.1016/j.cger.2009.07.007 PMC2797320 19944261

[3] Duprez DA. Angina in the Elderly. Eur Heart J. 1996;17(Suppl G):8-13. doi: 10.1093/eurheartj/17.suppl_g.8. 10.1093/eurheartj/17.suppl_g.8 8960449

[4] Sheifer SE, Gersh BJ, Yanez ND 3rd, Ades PA, Burke GL, Manolio TA. Prevalence, Predisposing Factors, and Prognosis of Clinically Unrecognized Myocardial Infarction in the Elderly. J Am Coll Cardiol. 2000;35(1):119-26. doi: 10.1016/s0735-1097(99)00524-0. 10.1016/s0735-1097(99)00524-0 10636269

[5] Vincenti G, Masci PG, Monney P, Rutz T, Hugelshofer S, Gaxherri M, et al. Stress Perfusion CMR in Patients with Known and Suspected CAD: Prognostic Value and Optimal Ischemic Threshold for Revascularization. JACC Cardiovasc Imaging. 2017;10(5):526-37. doi: 10.1016/j.jcmg.2017.02.006. 10.1016/j.jcmg.2017.02.006 28412420

[6] Jahnke C, Nagel E, Gebker R, Kokocinski T, Kelle S, Manka R, et al. Prognostic Value of Cardiac Magnetic Resonance Stress Tests: Adenosine Stress Perfusion and Dobutamine Stress Wall Motion Imaging. Circulation. 2007;115(13):1769-76. doi: 10.1161/CIRCULATIONAHA.106.652016. 10.1161/CIRCULATIONAHA.106.652016 17353441

[7] Lipinski MJ, McVey CM, Berger JS, Kramer CM, Salerno M. Prognostic Value of Stress Cardiac Magnetic Resonance Imaging in Patients with Known or Suspected Coronary Artery Disease: A Systematic Review and Meta-analysis. J Am Coll Cardiol. 2013;62(9):826-38. doi: 10.1016/j.jacc.2013.03.080. 10.1016/j.jacc.2013.03.080 PMC3863376 23727209

[8] Heitner JF, Kim RJ, Kim HW, Klem I, Shah DJ, Debs D, et al. Prognostic Value of Vasodilator Stress Cardiac Magnetic Resonance Imaging: A Multicenter Study With 48 000 Patient-Years of Follow-up. JAMA Cardiol. 2019;4(3):256-64. doi: 10.1001/jamacardio.2019.0035. 10.1001/jamacardio.2019.0035 PMC6439546 30735566

[9] Ashrafpoor G et al. Stress Cardiac Magnetic Resonance Imaging in Elderly Patients [abstract]. J Cardiovasc Magn Reson. 2011;13(Suppl 1):102. doi: 10.1186/1532-429X-13-S1-P102.

[10] Pezel T, Sanguineti F, Kinnel M, Hovasse T, Garot P, Unterseeh T, et al. Prognostic Value of Dipyridamole Stress Perfusion Cardiovascular Magnetic Resonance in Elderly Patients >75 years with Suspected Coronary Artery Disease. Eur Heart J Cardiovasc Imaging. 2021;22(8):904-11. doi: 10.1093/ehjci/jeaa193. 10.1093/ehjci/jeaa193 32756995

[11] Mancia G, Fagard R, Narkiewicz K, Redon J, Zanchetti A, Böhm M, et al. 2013 ESH/ESC Guidelines for the Management of Arterial Hypertension: The Task Force for the Management of Arterial Hypertension of the European Society of Hypertension (ESH) and of the European Society of Cardiology (ESC). Eur Heart J. 2013;34(28):2159-219. doi: 10.1093/eurheartj/eht151. 10.1093/eurheartj/eht151 23771844

[12] American Diabetes Association. Standards of Medical Care in Diabetes--2014. Diabetes Care. 2014;37(Suppl 1):S14-80. doi: 10.2337/dc14-S014. 10.2337/dc14-S014 24357209

[13] Stone NJ, Robinson JG, Lichtenstein AH, Merz CNB, Blum CB, Eckel RH, et al. 2013 ACC/AHA Guideline on the Treatment of Blood Cholesterol to Reduce Atherosclerotic Cardiovascular Risk in Adults: A Report of the American College of Cardiology/American Heart Association Task Force on Practice Guidelines. J Am Coll Cardiol. 2014;63(25 Pt B):2889-934. doi: 10.1016/j.jacc.2013.11.002. 10.1016/j.jacc.2013.11.002 24239923

[14] Montalescot G, Sechtem U, Achenbach S, Andreotti F, Arden C, Budaj A, et al. 2013 ESC Guidelines on the Management of Stable Coronary Artery Disease: The Task Force on the Management of Stable Coronary Artery Disease of the European Society of Cardiology. Eur Heart J. 2013;34(38):2949-3003. doi: 10.1093/eurheartj/eht296. 10.1093/eurheartj/eht296 23996286

[15] Grobner T, Prischl FC. Gadolinium and Nephrogenic Systemic Fibrosis. Kidney Int. 2007;72(3):260-4. doi: 10.1038/sj.ki.5002338. 10.1038/sj.ki.5002338 17507905

[16] Kramer CM, Barkhausen J, Bucciarelli-Ducci C, Flamm SD, Kim RJ, Nagel E. Standardized Cardiovascular Magnetic Resonance Imaging (CMR) Protocols: 2020 Update. J Cardiovasc Magn Reson. 2020;22(1):17. doi: 10.1186/s12968-020-00607-1. 10.1186/s12968-020-00607-1 PMC7038611 32089132

[17] Schulz-Menger J, Bluemke DA, Bremerich J, Flamm SD, Fogel MA, Friedrich MG, et al. Standardized Image Interpretation and Post-processing in Cardiovascular Magnetic Resonance - 2020 Update: Society for Cardiovascular Magnetic Resonance (SCMR): Board of Trustees Task Force on Standardized Post-Processing. J Cardiovasc Magn Reson. 2020;22(1):19. doi: 10.1186/s12968-020-00610-6. 10.1186/s12968-020-00610-6 PMC7066763 32160925

[18] Cerqueira MD, Weissman NJ, Dilsizian V, Jacobs AK, Kaul S, Laskey WK, et al. Standardized Myocardial Segmentation and Nomenclature for Tomographic Imaging of the Heart. A Statement for Healthcare Professionals from the Cardiac Imaging Committee of the Council on Clinical Cardiology of the American Heart Association. Circulation. 2002;105(4):539-42. doi: 10.1161/hc0402.102975. 10.1161/hc0402.102975 11815441

[19] Hicks KA, Mahaffey KW, Mehran R, Nissen SE, Wiviott SD, Dunn B, et al. 2017 Cardiovascular and Stroke Endpoint Definitions for Clinical Trials. J Am Coll Cardiol. 2018;71(9):1021-34. doi: 10.1016/j.jacc.2017.12.048. 10.1016/j.jacc.2017.12.048 29495982

[20] Greenwood JP, Maredia N, Younger JF, Brown JM, Nixon J, Everett CC, et al. Cardiovascular Magnetic Resonance and Single-photon Emission Computed Tomography for Diagnosis of Coronary Heart Disease (CE-MARC): A Prospective Trial. Lancet. 2012;379(9814):453-60. doi: 10.1016/S0140-6736(11)61335-4. 10.1016/S0140-6736(11)61335-4 PMC3273722 22196944

[21] Schwitter J, Wacker CM, Wilke N, Al-Saadi N, Sauer E, Huettle K, et al. MR-IMPACT II: Magnetic Resonance Imaging for Myocardial Perfusion Assessment in Coronary Artery Disease Trial: Perfusion-cardiac Magnetic Resonance vs. Single-photon Emission Computed Tomography for the Detection of Coronary Artery Disease: A Comparative Multicentre, Multivendor Trial. Eur Heart J. 2013;34(10):775-81. doi: 10.1093/eurheartj/ehs022. 10.1093/eurheartj/ehs022 22390914

[22] Cicala S, de Simone G, Roman MJ, Best LG, Lee ET, Wang W, et al. Prevalence and Prognostic Significance of Wall-motion Abnormalities in Adults without Clinically Recognized Cardiovascular Disease: The Strong Heart Study. Circulation. 2007;116(2):143-50. doi: 10.1161/CIRCULATIONAHA.106.652149. 10.1161/CIRCULATIONAHA.106.652149 17576870

[23] Grothues F, Moon JC, Bellenger NG, Smith GS, Klein HU, Pennell DJ. Interstudy Reproducibility of Right Ventricular Volumes, Function, and Mass with Cardiovascular Magnetic Resonance. Am Heart J. 2004;147(2):218-23. doi: 10.1016/j.ahj.2003.10.005. 10.1016/j.ahj.2003.10.005 14760316

[24] Bellenger NG, Burgess MI, Ray SG, Lahiri A, Coats AJ, Cleland JG, et al. Comparison of Left Ventricular Ejection Fraction and Volumes in Heart Failure by Echocardiography, Radionuclide Ventriculography and Cardiovascular Magnetic Resonance; are they Interchangeable? Eur Heart J. 2000;21(16):1387-96. doi: 10.1053/euhj.2000.2011. 10.1053/euhj.2000.2011 10952828

[25] Kim RJ, Fieno DS, Parrish TB, Harris K, Chen EL, Simonetti O, et al. Relationship of MRI Delayed Contrast Enhancement to Irreversible Injury, Infarct Age, and Contractile Function. Circulation. 1999;100(19):1992-2002. doi: 10.1161/01.cir.100.19.1992. 10.1161/01.cir.100.19.1992 10556226

[26] Mahrholdt H, Wagner A, Judd RM, Sechtem U, Kim RJ. Delayed Enhancement Cardiovascular Magnetic Resonance Assessment of Non-ischaemic Cardiomyopathies. Eur Heart J. 2005;26(15):1461-74. doi: 10.1093/eurheartj/ehi258. 10.1093/eurheartj/ehi258 15831557

[27] Ponikowski P, Voors AA, Anker SD, Bueno H, Cleland JGF, Coats AJS, et al. 2016 ESC Guidelines for the Diagnosis and Treatment of Acute and Chronic Heart Failure: The Task Force for the Diagnosis and Treatment of Acute and Chronic Heart Failure of the European Society of Cardiology (ESC)Developed with the Special Contribution of the Heart Failure Association (HFA) of the ESC. Eur Heart J. 2016;37(27):2129-200. doi: 10.1093/eurheartj/ehw128. 10.1093/eurheartj/ehw128 27206819

[28] Krittayaphong R, Saiviroonporn P, Boonyasirinant T, Udompunturak S. Prevalence and Prognosis of Myocardial Scar in Patients with Known or Suspected Coronary Artery Disease and Normal Wall Motion. J Cardiovasc Magn Reson. 2011;13(1):2. doi: 10.1186/1532-429X-13-2. 10.1186/1532-429X-13-2 PMC3022594 21211011

[29] Kwong RY, Korlakunta H. Diagnostic and Prognostic Value of Cardiac Magnetic Resonance Imaging in Assessing Myocardial Viability. Top Magn Reson Imaging. 2008;19(1):15-24. doi: 10.1097/RMR.0B013e31817d550c. 10.1097/RMR.0B013e31817d550c 18690157

[30] Kim HW, Klem I, Shah DJ, Wu E, Meyers SN, Parker MA, et al. Unrecognized non-Q-wave Myocardial Infarction: Prevalence and Prognostic Significance in Patients with Suspected Coronary Disease. PLoS Med. 2009;6(4):e1000057. doi: 10.1371/journal.pmed.1000057. 10.1371/journal.pmed.1000057 PMC2661255 19381280

[31] Kehl DW, Farzaneh-Far R, Na B, Whooley MA. Prognostic Value of Electrocardiographic Detection of Unrecognized Myocardial Infarction in Persons with Stable Coronary Artery Disease: Data from the Heart and Soul Study. Clin Res Cardiol. 2011;100(4):359-66. doi: 10.1007/s00392-010-0255-2. 10.1007/s00392-010-0255-2 PMC3062762 21103882

[32] Pride YB, Piccirillo BJ, Gibson CM. Prevalence, Consequences, and Implications for Clinical Trials of Unrecognized Myocardial Infarction. Am J Cardiol. 2013;111(6):914-8. doi: 10.1016/j.amjcard.2012.11.042. 10.1016/j.amjcard.2012.11.042 23276472

[33] Nordenskjöld AM, Hammar P, Ahlström H, Bjerner T, Duvernoy O, Lindahl B. Unrecognized Myocardial Infarction Assessed by Cardiac Magnetic Resonance Imaging is Associated with Adverse Long-term Prognosis. PLoS One. 2018;13(7):e0200381. doi: 10.1371/journal.pone.0200381. 10.1371/journal.pone.0200381 PMC6034881 29979788

