# Supplementary material for: Valor Prognóstico da Imagem de Ressonância Magnética Cardíaca de Perfusão em Estresse com Adenosina em Idosos com Doença Arterial Coronariana Conhecida ou Suspeita
Source: Arq Bras Cardiol. 2022 Jul 7;119(1):97–106. [Article in Portuguese] doi: 10.36660/abc.20210530 (PMC9352122; doi:10.36660/abc.20210530)
Supplement: Translation

# Translation: Prognostic Value of Adenosine Stress Perfusion Cardiac Magnetic Resonance Imaging in Older Adults with Known or Suspected Coronary Artery Disease

**Authors:** Yodying Kaolawanich, Thananya Boonyasirinant

**Affiliations:** 1 Bangkok Thailand Division of Cardiology, Department of Medicine, Faculty of Medicine Siriraj Hospital, Mahidol University, Bangkok – Thailand

**Keywords:** Adenosine, Cardiac Magnetic Resonance Imaging, Coronary Artery Disease, Elderly, Stress Test

## Abstract

**Background:**

There is limited data on the prognostic value of stress cardiac magnetic resonance (CMR) in older adults.

**Objective:**

To determine the prognostic value of adenosine stress CMR in older individuals with known or suspected coronary artery disease (CAD).

**Methods:**

Between 2010 and 2015, consecutive patients aged 65 years or older referred for adenosine stress CMR were followed for the occurrence of severe cardiac events (cardiac death and nonfatal myocardial infarction) and major adverse cardiovascular events (MACE) that also included hospitalization for heart failure and ischemic stroke. Univariate and multivariate analyses were performed to determine the prognostic value of myocardial ischemia, with p-value <0.05 considered statistically significant.

**Results:**

After a mean follow-up period of 50.4 months in 324 patients (48% male, 73±7 years), 21 severe cardiac events and 52 MACE occurred. Patients with myocardial ischemia (n=99) had significantly higher rates of severe cardiac events (HR 5.25 [95% CI 2.11-13.04], p<0.001) and MACE (HR 3.01 [95% CI 1.75-5.20], p<0.001) than those without ischemia. Multivariable analysis determined ischemia as an independent predictor of severe cardiac events (HR 3.14 [95% CI 1.22-8.07], p=0.02) and MACE (HR 1.91 [95%CI 1.02-3.59], p=0.04). Ischemia provided an incremental prognostic value over clinical factors and left ventricular ejection fraction for predicting severe cardiac events and MACE (p<0.01 for both). No severe adverse events occurred during or immediately after CMR examinations.

**Conclusion:**

Adenosine stress CMR is safe and has prognostic value in older adults with known or suspected CAD.

## Introduction

Aging is associated with diffuse changes throughout the cardiovascular system. The prevalence and severity of coronary artery disease (CAD) increase progressively with age in both men and women.^1^ In developed countries, approximately two-thirds of all myocardial infarctions (MI) occur in people over 65 years old.^2^ The elderly are more likely to present with atypical symptoms such as exertional shortness of breath or fatigue rather than typical angina.^3^ The prevalence of silent myocardial ischemia and unrecognized myocardial infarction (MI) is also significantly higher in the elderly and has prognostic value.^4^ Older patients also tend to be at increased risk for complications including heart failure, arrhythmias, bleeding, and death in the setting of cardiac procedures, such as percutaneous coronary intervention or cardiac surgery. Therefore, diagnosis and risk stratification of CAD in elderly patients are critically important.

Testing for ischemia in elderly patients is challenging. Exercise testing is less feasible in older adults due to lower exercise capacity and comorbidities, as well as baseline electrocardiographic (ECG) abnormalities that limit ischemic assessments. Cardiac magnetic resonance (CMR) provides a comprehensive assessment of CAD with very high accuracy. CMR can assess global and regional ventricular function, myocardial ischemia, and infarction in a single study. Moreover, pharmacological stress CMR offer strong evidence for the prognosis, including mortality in patients with known or suspected CAD.^5–8^

Previous data have shown that stress perfusion CMR performed in elderly patients is safe and well-tolerated.^9,10^ A recent study reported the prognostic value of dipyridamole stress perfusion CMR in elderly patients with suspected CAD.^10^ Adenosine is most often used for stress perfusion CMR in clinical practice. However, prognostic data of adenosine stress CMR in elderly patients remain limited.

The objective of this study was to determine the prognostic value of adenosine stress CMR in older adults with known or suspected CAD.

## Methods

### Study population

Consecutive patients older than 65 years with known or suspected CAD, who were referred for adenosine stress CMR from January 2010 to December 2015 at our outpatient center were enrolled. Detailed medical history was collected on the same day of CMR examination. History of hypertension, diabetes mellitus, hyperlipidemia, CAD, and stroke was defined by recent guidelines.^11–14^

Exclusion criteria included (i) known non-ischemic cardiomyopathy (e.g., hypertrophic, dilated, or infiltrative), (ii) incomplete CMR examination, (iii) poor CMR images, and (v) lack of follow-up data. The institutional ethics committee approved this retrospective study and waived the need for additional written informed consent.

Concern has been expressed regarding the association of gadolinium use with the development of nephrogenic systemic fibrosis in patients with severe kidney injury, especially in the elderly. Patients who had glomerular filtration rate <30 mL/min/1.73m^2^ did not undergo a contrast-enhanced CMR examination and were not included in this study.^15^

### CMR protocol

The CMR study was performed to assess cardiac function, myocardial perfusion, and late gadolinium enhancement (LGE) using a 1.5 Tesla Philips Achieva XR scanner (Philips Medical Systems, Best, The Netherlands).

The cardiac functional study was performed by acquiring the images using the steady-state free precession (SSFP) technique in a vertical long axis, 2-chamber, 4-chamber, and multiple slice short-axis views. Parameters for cardiac function were echo time (TE) 1.8 milliseconds (ms), repetitive time (TR) 3.7 ms, number of excitations 2, field of view (FOV) 390 x 312 mm, matrix 256 x 240, reconstruction pixels 1.52 x 1.21, slide thickness 8 mm, and flip angle of 70 degrees.

The myocardial first-pass perfusion study was performed by injecting 0.05 mmol/kg of gadolinium contrast agent (Magnevist, Bayer Schering Pharma, Berlin, Germany) at a rate of 4 mL/s immediately after a 4-minute infusion of 140 mcg/kg/min of adenosine.^16^ If after 3 minutes of continuous infusion at the standard rate, the hemodynamic response to adenosine was inadequate (heart rate increase <10 beats/min or systolic blood pressure decrease <10 mmHg, with minimal or no reported side effects from the patient), then the infusion rate was increased up to 210 mcg/kg/min for a further 2 minutes.^16^ Three short-axis slices of basal, mid, and apical left ventricular (LV) levels were acquired using an ECG-triggered, SSFP, inversion-recovery, single-shot, turbo gradient-echo sequence. Image parameters were TE 1.32 ms, TR 2.6 ms, flip angle 50 degrees, slide thickness 8 mm, FOV 270 mm, and reconstructed FOV 320 mm.

LGE images were acquired approximately 10 minutes after an additional bolus of gadolinium (0.1 mmol/kg, rate 4 mL/s) by the 3D segmented-gradient-echo inversion-recovery sequence. LGE images were acquired in multiple short-axis slices at levels similar to the functional images, long axis, 2-chamber and 4-chamber view. Parameters for LGE study were TE 1.25 ms, TR 4.1 ms, flip angle 15 degrees, FOV 303 x 384 mm, matrix 240 x 256, in-plane resolution 1.26 x 1.5 mm, slice thickness 8 mm and 1.5 sensitivity-encoding factor.

### Image analysis

Standard LV volumes, mass, and ejection fraction (EF) were quantitatively measured from the stack of short-axis SSFP cine images.

The perfusion and LGE images were analyzed using visual assessment and consensus by two CMR-trained physicians blinded to the clinical and follow-up data. Perfusion images were read, and each of the 16 segments was visualized (segment-17 at the apex was not visualized). Inducible ischemia was defined as a subendocardial perfusion defect that (i) persisted beyond peak myocardial enhancement and for several RR intervals, (ii) was more than two pixels wide, (iii) followed one or more coronary arteries, and (iv) showed absence of LGE in the same segment.^10,17^ Dark-banding artefacts were recorded if an endocardial dark band appeared at the arrival of contrast in the LV cavity before contrast arrival in the myocardium.^17^ LGE images were also analyzed using visual assessment. LGE was considered present only if confirmed on both the short-axis and at least one other orthogonal plane.^17^ The total number of LGE segments was calculated using the American Heart Association 17-segment model.^18^

### Clinical follow-up

Follow-up data were collected from clinical visits and medical records. Clinical event adjudication was completely blinded to clinical and CMR data. Patients were followed for severe cardiac events and major adverse cardiovascular events (MACE). Severe cardiac events were defined as the composite outcomes of cardiac mortality and nonfatal MI.^19^ MACE was defined as the composite outcomes of cardiac mortality, nonfatal MI, hospitalization for heart failure, and ischemic stroke.

### Statistical analysis

Statistical analyses were performed using IBM SPSS Statistics for Windows, version 20.0 (IBM Corp., Armonk, NY, USA). Continuous variables with normal distribution were presented as mean ± standard deviation (SD). The normality of variable distribution was assessed by the Kolmogorov-Smirnov test. Categorical variables were presented as absolute numbers and percentages. Differences between patients with and without myocardial ischemia in terms of clinical baseline and CMR characteristics were compared using the Student’s unpaired t-test for continuous variables, and the chi-square test or Fisher’s exact test for categorical variables, as appropriate.

Composite outcomes between patients with and without myocardial ischemia were estimated using the Kaplan-Meier method and compared with the log-rank test. To analyze the predictors of severe cardiac events and MACE, a Cox-regression analysis was performed to assess univariable predictors from baseline characteristics and CMR parameters. Variables with p-value <0.05 in the univariable analysis were entered into the multivariable analysis. Two multivariable models were developed to assess the prognostic value of myocardial ischemia. First, ischemia was included as a categorical variable (presence or absence). Second, ischemia was included as a continuous variable (per-segment extent).

To assess the incremental prognostic values of significant predictors, global chi-square values were calculated after adding predictors in the following order: clinical, LVEF, ischemia, and LGE.

The hazard ratios (HRs) and 95% confidence intervals (CIs) of the outcomes were calculated, with a p-value <0.05 considered statistically significant.

## Results

A total of 327 patients were enrolled, with three excluded due to loss of follow-up data. No patients were excluded because of poor image quality, and 324 were included in the final analysis. Table 1 summarizes the clinical data of the patient population. The average age was 73±7 years. Forty-six patients had known CAD, and 6 had previous MI. The overall study cohort had mean LVEF of 68.8±13.8%.

**Table 1:** Clinical characteristics of patients with and without myocardial ischemia

	Total	Ischemia Present	Ischemia Absent	p-value
	(n=324)	(n=99)	(n=225)	
Male gender	156 (48.1)	55 (55.6)	101 (44.9)	0.08
Age, years	72.7 ± 7.4	72.9 ± 7.7	72.6 ± 7.3	0.73
Body mass index, kg/m^2^	26.5 ± 4.2	25.8 ± 3.9	26.9 ± 4.2	**0.03**
Systolic blood pressure, mmHg	138.8 ± 18.9	142.2 ± 19.3	137.3 ± 18.7	**0.03**
Diastolic blood pressure, mmHg	72.8 ± 11.5	71.9 ± 12.1	73.2 ± 11.2	0.33
Heart rate, bpm	76.9 ± 13.1	76.2 ± 12.8	77.2 ± 13.3	0.52
Hypertension	289 (89.2)	87 (87.8)	202 (89.8)	0.61
Diabetes mellitus	188 (58.0)	57 (57.6)	131 (58.2)	0.91
Hyperlipidemia	231 (71.3)	74 (74.8)	157 (69.8)	0.36
Stable coronary artery disease	46 (14.2)	28 (28.3)	18 (8.0)	**<0.001**
Previous myocardial infarction	6 (1.9)	5 (5.1)	1 (0.4)	**0.01**
Prior revascularization	14 (4.3)	8 (8.1)	6 (2.7)	**0.04**
History of typical angina	31 (9.6)	15 (15.2)	16 (7.1)	**0.02**
History of heart failure	23 (7.1)	9 (9.1)	14 (6.2)	0.35
Stroke	16 (4.9)	4 (4.0)	12 (5.3)	0.78
Current smoking	37 (11.4)	22 (22.2)	15 (6.7)	**<0.001**
**Medications**				
ACEI or ARB	148 (45.7)	50 (50.5)	98 (43.6)	0.25
Antiplatelet	153 (47.2)	60 (60.6)	93 (41.3)	**0.001**
Beta-blocker	151 (46.6)	47 (47.5)	104 (46.2)	0.84
Calcium channel blocker	111 (34.3)	35 (35.4)	76 (33.8)	0.78
Nitrate	49 (15.1)	25 (25.3)	24 (10.7)	**0.001**
Statin	156 (48.2)	51 (51.5)	105 (46.7)	0.42
**CMR**				
Left atrial diameter, mm	32.9 ± 4.0	33.6 ± 4.1	32.6 ± 3.9	0.05
LV mass index, g/m^2^	51.9 ± 16.8	59.0 ± 18.8	48.9 ± 14.8	**<0.001**
LVEDV index, ml/m^2^	74.7 ± 24.4	82.1 ± 29.0	71.5 ± 21.4	**<0.001**
LVESV index, ml/m^2^	25.7 ± 22.9	32.2 ± 29.9	22.8 ± 18.3	**<0.001**
LVEF, %	68.8 ± 13.8	65.1 ± 17.5	70.5 ± 11.5	**0.001**
Presence of LGE	67 (20.7)	45 (45.5)	22 (9.8)	**<0.001**
Average numbers of segments with LGE	4.1 ± 2.5	4.3 ± 2.6	3.6 ± 2.4	0.16

Values are number (percentages) or mean ± SD. **Bold** values are <0.05. ACEI: angiotensin-converting enzyme inhibitor; ARB: angiotensin II receptor blocker; CMR: cardiac magnetic resonance; EDV: end diastolic volume; ESV: end systolic volume; EF: ejection fraction; LGE: late gadolinium enhancement; LV: left ventricular.

Myocardial ischemia was detected in 99 patients (31%), with the average number of ischemic segments of 6.9±3.9. Sixty-seven had LGE, and all showed a CAD pattern (subendocardial or transmural LGE). Among 67 patients that had LGE, 3 had a history of MI. Thus, 64 patients (19.7%) had LGE without a history of MI (‘unrecognized MI’).

Patients with myocardial ischemia had a greater LV mass index, lower LVEF, and higher prevalence of LGE than those without ischemia. Patients with ischemia were also more likely to have a history of CAD or MI and be on antiplatelet and nitrate therapy.

No patient died during or shortly after CMR, while one patient had mild heart failure requiring adjustment of diuretics without hospital admission. Two patients experienced angina that rapidly resolved with sublingual nitrate use. No cases of acute MI or strokes were recorded during or immediately after CMR. The main minor adverse events included headache, nausea, chest discomfort, dyspnea, and transient blood pressure drop.

During the average follow-up period of 50.4±19.2 months, 21 severe cardiac events and 52 MACE occurred. Table 2 depicts the cardiovascular events in patients with and without ischemia. The Kaplan-Meier curves of both groups are shown in Figure 1. Patients with myocardial ischemia had significantly higher rates of severe cardiac events (annual events rate 3.8% *versus* 0.7%, p<0.001) and MACE (annual event rate 7.9% *versus* 2.7%, p<0.001) than those without ischemia.

**Table 2:** Patients’ outcomes

	Total	Ischemia Present	Ischemia Absent	HR (95% CI)	p Value
All-cause mortality	18 (5.6)	10 (10.1)	8 (3.6)	3.13 (1.23, 7.94)	**0.02**
Cardiac mortality	8 (2.5)	6 (6.1)	2 (0.9)	7.59 (1.53, 37.66)	**0.01**
Nonfatal myocardial infarction	18 (5.6)	12 (12.1)	6 (2.7)	5.22 (1.95, 13.94)	**0.001**
Hospitalization for heart failure	31 (9.6)	16 (16.2)	15 (6.7)	2.81(1.38, 5.70)	**0.004**
Ischemic stroke	9 (2.8)	3 (3.0)	6 (2.7)	1.31 (0.32, 5.25)	0.70
Severe cardiac events^a^	21 (6.5)	14 (14.1)	7 (3.1)	5.25 (2.11, 13.04)	**<0.001**
MACE^b^	52 (16.0)	27 (27.3)	25 (11.1)	3.01 (1.75, 5.20)	**<0.001**

Severe cardiac events=composite outcomes of cardiac mortality and nonfatal myocardial infarction. MACE: composite outcomes of cardiac mortality, nonfatal myocardial infarction, hospitalized for heart failure, and ischemic stroke.

a Five patients had two events (nonfatal myocardial infarction and cardiac mortality).

b Nine patients had more than one event (six patients had two events, one patient had three events, and two patients had four events). Values represent the number of patients (percentages). **Bold** values are <0.05. CI: confidence interval; HR: hazard ratio; MACE: major adverse cardiovascular events.

**Figure 1:** Kaplan-Meier curves for the incidence of severe cardiac events (A) and MACE (B). HR: hazard ratio; MACE: major adverse cardiovascular events.

Univariable and multivariable analyses for the prediction of severe cardiac events and MACE are shown in Tables 3 and 4, respectively. The number of patients and events were limited; therefore, to avoid the potential for overfitting, only the most significant predictors from univariable analysis were included in any multivariable model.

**Table 3:** Predictors of severe cardiac events

	Univariable Analysis		Multivariable Analysis			
			Model 1^a^		Model 2^b^	
	HR (95% CI)	P-Value	HR (95% CI)	p Value	HR (95% CI)	p-value
Male gender	1.26 (0.53, 2.97)	0.59				
Age, years	1.01 (0.95, 1.07)	0.70				
Body mass index, kg/m^2^	0.90 (0.81, 1.01)	0.08				
Systolic blood pressure	0.99 (0.97, 1.02)	0.63				
Diastolic blood pressure	0.98 (0.94, 1.02)	0.33				
Heart rate, bpm	1.01 (0.97, 1.04)	0.71				
Hypertension	2.57 (0.34, 19.17)	0.36				
Diabetes mellitus	1.21 (0.51, 2.89)	0.67				
Hyperlipidemia	1.06 (0.39, 2.92)	0.90				
Stable coronary artery disease	2.26 (0.82, 6.19)	0.11				
Previous myocardial infarction	9.36 (2.75, 31.81)	**<0.001**	6.70 (1.83, 24.49)	**0.004**	5.90 (1.52, 22.93)	**0.01**
History of typical angina	2.80 (1.02, 7.65)	**0.04**				
History of heart failure	2.78 (0.93, 8.30)	0.07				
Stroke	0.05 (0.00-177.4)	0.46				
Current smoking	1.82 (0.61, 5.41)	0.28				
ACEI or ARB	1.11 (0.46, 2.60)	0.82				
Antiplatelet	2.09 (0.84, 5.20)	0.11				
Beta-blocker	1.15 (0.48, 2.71)	0.75				
Calcium channel blocker	0.96 (0.38, 2.38)	0.94				
Nitrate	3.03 (1.25, 7.33)	**0.01**				
Statin	1.46 (0.61, 3.47)	0.39				
Left atrial diameter, mm	1.16 (1.06, 1.27)	**0.002**				
LV mass index, g/m^2^	1.03 (1.02, 1.05)	**<0.001**	1.04 (1.02, 1.05)	**0.001**	1.03 (1.02, 1.05)	**0.001**
LVEDV index, ml/m^2^	1.02 (1.01, 1.03)	**<0.001**				
LVESV index, ml/m^2^	1.02 (1.01, 1.03)	**0.001**				
LVEF, %	0.96 (0.94, 0.99)	**0.01**				
Presence of myocardial ischemia	5.25 (2.11, 13.04)	**<0.001**	3.14 (1.22, 8.07)	**0.02**	**-**	**-**
Ischemia extent, per 1 segment	1.17 (1.09, 1.26)	**<0.001**	-	**-**	1.11 (1.02, 1.20)	**0.01**
Presence of LGE	4.97 (2.11, 11.73)	**<0.001**

a Myocardial ischemia was included as a categorical variable (presence or absence).

b Myocardial ischemia was included as a continuous variable (per-segment extent). **Bold** values are <0.05. ACEI: angiotensin-converting enzyme inhibitor; ARB: angiotensin II receptor blocker; CMR: cardiac magnetic resonance; EDV: end diastolic volume; ESV: end systolic volume; EF: ejection fraction; LGE: late gadolinium enhancement; LV: left ventricular.

**Table 4:** Predictors of major adverse cardiovascular events

	Univariable Analysis		Multivariable Analysis			
			Model 1^a^		Model 2^b^	
	HR (95% CI)	p Value	HR (95% CI)	p Value	HR (95% CI)	p-value
Male gender	1.15 (0.67, 1.99)	0.61				
Age, years	1.05 (1.01, 1.08)	**0.02**				
Body mass index, kg/m^2^	0.98 (0.92, 1.05)	0.60				
Systolic blood pressure	0.99 (0.98, 1.01)	0.43				
Diastolic blood pressure	0.97 (0.95, 0.99)	**0.02**				
Heart rate, bpm	1.01 (0.99, 1.03)	0.30				
Hypertension	2.11 (0.66, 6.78)	0.21				
Diabetes mellitus	1.21 (0.70, 2.11)	0.50				
Hyperlipidemia	1.17 (0.61, 2.23)	0.64				
Stable coronary artery disease	1.58 (0.77, 3.24)	0.22				
Previous myocardial infarction	6.13 (2.21, 17.06)	**0.001**				
History of typical angina	1.43 (0.64, 3.17)	0.38				
History of heart failure	3.70 (1.90, 7.20)	**<0.001**	3.50 (1.79, 6.82)	**0.001**	3.32 (1.70, 6.50)	**0.001**
Stroke	1.15 (0.36, 3.70)	0.81				
Current smoking	1.62 (0.79, 3.33)	0.19				
ACEI or ARB	1.23 (0.71, 2.11)	0.46				
Antiplatelet	1.57 (0.90, 2.73)	0.11				
Beta blocker	1.02 (0.59, 1.77)	0.93				
Calcium channel blocker	0.69 (0.37, 1.27)	0.24				
Nitrate	1.87 (1.01, 3.45)	**0.04**				
Statin	1.19 (0.69, 2.05)	0.53				
Left atrial diameter, mm	1.13 (1.06, 1.20)	**<0.001**				
LV mass index, g/m^2^	1.03 (1.02, 1.04)	**<0.001**				
LVEDV index, ml/m^2^	1.02 (1.01, 1.03)	**<0.001**				
LVESV index, ml/m^2^	1.02 (1.01, 1.03)	**<0.001**				
LVEF, %	0.97 (0.95, 0.98)	**<0.001**				
Presence of myocardial ischemia	3.01 (1.75, 5.20)	**<0.001**	1.91 (1.02, 3.59)	**0.04**	-	-
Ischemia extent, per 1 segment	1.11 (1.06, 1.17)	**<0.001**	-	-	1.08 (1.01, 1.14)	**0.02**
Presence of LGE	3.70 (2.13, 6.43)	**<0.001**	2.64 (1.39, 4.99)	**0.003**	2.86 (1.58, 5.17)	**0.001**

a Myocardial ischemia was included as a categorical variable (present or absent).

b Myocardial ischemia was included as a continuous variable (per-segment extent). **Bold** values are <0.05. ACEI: angiotensin-converting enzyme inhibitor; ARB: angiotensin II receptor blocker; CMR: cardiac magnetic resonance; EDV: end diastolic volume; ESV: end systolic volume; EF: ejection fraction; MACE: major adverse cardiovascular events; LGE: late gadolinium enhancement; LV: left ventricular.

The most significant predictors identified by the univariable analysis for severe cardiac events were previous MI, LV mass index, LV end-diastolic volume index, myocardial ischemia, and LGE (p<0.001 for all). A history of heart failure, left atrial diameter, LV mass index, LVEF, myocardial ischemia, and LGE were the most significant predictors of MACE (p<0.001 for all).

Multivariable analyses showed that previous MI, LV mass index, and myocardial ischemia were independent predictors of severe cardiac events. For MACE, history of heart failure, myocardial ischemia, and LGE were independent predictors. Note that both the presence of myocardial ischemia (model 1) and the number of ischemic segments (model 2) were independent predictors for severe cardiac events and MACE.

Figure 2 shows the incremental prognostic values of clinical and CMR data for the prediction of severe cardiac events and MACE. When the prognosis was assessed in a hierarchical manner (clinical variables only, clinical+LVEF, clinical+LVEF+ischemia, and clinical+LVEF+ischemia+LGE), the presence of myocardial ischemia demonstrated an incremental prognostic value over clinical variables and LVEF for both severe cardiac events (Figure 2A) and MACE (Figure 2B). Adding LGE provided a further incremental prognostic value for MACE (Figure 2B). However, LGE did not show an incremental prognostic value over ischemia for severe cardiac events (Figure 2A).

**Figure 2:** Incremental prognostic value of LVEF, myocardial ischemia, and LGE for severe cardiac events (A) and MACE (B). Clinical=age, male gender, previous myocardial infarction, and history of heart failure. LGE: late gadolinium enhancement; LVEF: left ventricular ejection fraction; MACE: major adverse cardiovascular events.

Eighteen patients died during the follow-up. Ten patients died from non-cardiac causes (e.g., malignancy). Patients with myocardial ischemia had a significantly higher rate of all-cause mortality than those without ischemia (Table 2). However, there was no significant difference between patients with and without ischemia regarding the non-cardiac mortality rate (HR 1.66, 95% CI 0.47-5.88, p=0.44).

## Discussion

Our results demonstrated that myocardial ischemia using adenosine stress perfusion CMR was a strong and independent predictor of severe cardiac events and MACE in older adults with known or suspected CAD. Adenosine stress CMR was also feasible and safe in this population.

Most cardiovascular diseases, including CAD, increase in prevalence and severity with age. Diagnosis, risk stratification, and treatment of CAD in older patients remain challenging. Stable CAD manifests differently in the elderly, with exertional dyspnea, fatigue, and abdominal discomfort as the most common presentations.^3^ Aging and comorbidities limit exercise capacity; therefore, the ECG treadmill testing, and exercise echocardiography are impractical for this population. Pharmacological stress cardiac imaging, such as nuclear perfusion imaging and CMR are the preferred modalities; however, recent data has revealed limited accuracy of nuclear perfusion imaging compared to CMR. Data from large multicenter studies suggested that CMR had greater sensitivity than nuclear perfusion imaging for CAD detection in both males and females.^20,21^ Unlike nuclear perfusion imaging, CMR does not expose patients to ionizing radiation and offers both accuracy and safety.

Global and regional myocardial function is a well-known predictor of disease severity and prognosis.^22^ CMR is considered the gold standard for the assessment of global ventricular function and a good tool for the assessment of regional ventricular function.^23,24^ The elderly have a higher prevalence of lung diseases, such as chronic obstructive pulmonary disease and this may limit the assessment by echocardiography due to a poor echocardiographic window. CMR can assess cardiac function without the limitation of the cardiac plane, and also assess endocardial and epicardial borders without geometrical assumptions. Elderly patients may be more vulnerable to adverse events during or after CMR (e.g., arrhythmia or hypotension) due to the high prevalence of comorbidities. The applicability and safety of stress CMR were determined in patients older than 70 years, with results showing that stress CMR performed in elderly patients was safe and well-tolerated.^9,10^ Our results confirmed that adenosine stress CMR was safe in older adults without serious adverse events such as death, acute MI, or stroke during or immediately after CMR examinations.

Numerous studies have demonstrated the prognostic value of CMR in patients with known or suspected CAD.^5–8^ However, the mean age of patients in these studies was 60-65 years, with no specific assessment of the elderly. Pezel et al. reported on the prognostic value of dipyridamole stress perfusion CMR in 754 elderly patients aged over 75 with suspected CAD.^10^ In their study, 20% of the patients showed evidence of inducible ischemia, while 9.4% had LGE. The authors determined that the presence of myocardial ischemia was associated with the occurrence of MACE, including cardiac death and nonfatal MI.^10^ Our study, which included patients with known stable CAD and previous MI, found that 30.5% had inducible ischemia and 20.7% had LGE. The prevalence of myocardial ischemia in our study was comparable with previous reports that included patients with known CAD.^5,7^ Similarly, patients with inducible ischemia in our study demonstrated lower LVEF and higher prevalence of LGE than those without myocardial ischemia.^5–7^

Our results indicated that patients with inducible ischemia had significantly higher rates of severe cardiac events and MACE than those without ischemia. Myocardial ischemia was also an independent predictor of severe cardiac events and MACE. In contrast, patients without myocardial ischemia had a significantly lower risk for cumulative events (<1% per year for severe cardiac events). These findings agreed with those by Pezel et al.^10^

LGE is a well-validated method for detecting myocardial scars and fibrosis.^25^ Specific scar patterns corresponding to MI and various non-ischemic cardiomyopathy are diagnostically useful.^25,26^ Recent guidelines have highlighted the importance of myocardial fibrosis imaging by CMR.^14,27^ A significant proportion of patients with stable CAD have normal LV systolic function. The presence of LGE also demonstrated its prognostic value in patients with normal LVEF and wall motion.^28^ Similarly to our study, LV systolic function was preserved. LGE was detected in 20.7% of patients and was an independent predictor of MACE. Moreover, given the very small proportion of patients with a history of MI (< 2%), our data also demonstrated a compatible prevalence of ‘unrecognized MI’ (19.7%) when compared to previous data.^3,29–33^ Unrecognized MI is not an uncommon condition, with a prevalence of approximately 10-40% of patients with known or suspected CAD.^3,29–33^ LGE-CMR has improved the detection of small lesions due to MI (as little as 1 g), which do not give rise to Q-waves on the ECG.^29,30,33^ Additionally, recent studies consistently demonstrated that unrecognized MI using LGE-CMR was independently associated with an increased risk of cardiovascular events.^29,30,33^

### Limitations

Several limitations of our study should be considered. Firstly, the study methodology was retrospective and, therefore, some confounding factors could not be totally eliminated. Secondly, our stress protocol acquired only three short-axis slices to detect myocardial ischemia and may have underestimated perfusion defects in some small areas (compared to four or five short-axis slices). Thirdly, our study had a relatively low event rate, while some degree of overfitting may have occurred in the multivariable analyses. Finally, we did not provide the information regarding the adequacy of medical therapy after stress CMR that might affect the prognosis.

## Conclusions

Adenosine stress CMR is safe and shows prognostic value in older adults with known or suspected CAD.
